# Ventricular size on term magnetic resonance imaging in extremely preterm infants with and without germinal matrix-intraventricular haemorrhage

**DOI:** 10.1007/s00247-025-06508-8

**Published:** 2026-01-22

**Authors:** Maria Olsen Fossmark, Vasileios G. Xydis, Maria I. Argyropoulou, Loukas G. Astrakas, Hannah Bakøy, Mariann Bentsen, Derk Avenarius, Nils Thomas Songstad, Stein Magnus Aukland, Karen Rosendahl

**Affiliations:** 1 UiT The Arctic University of Norway, Tromsø, Norway; 2 University Hospital of North Norway, Tromsø, Norway; 3 University Hospital of Ioannina, Ioannina, Greece; 4 University of Ioannina, Ioannina, Greece; 5 University of Ioannina, Ioannina, Greece; 6 Haukeland University Hospital, Bergen, Norway; 7 University Hospital of North Norway, Tromsø, Norway; 8 UiT The Arctic University of Norway, Tromsø, Norway; 9 Haukeland University Hospital, Bergen, Norway; 10 UiT The Arctic University of Norway, 6050 Langnes, 9037 Tromsø, Norge

**Keywords:** Haemorrhage, Infant, extremely premature, Lateral ventricles, Magnetic resonance imaging, Prognosis, Reference values

## Abstract

**Background:**

Cerebral magnetic resonance imaging (MRI) at term-equivalent age can provide prognostic information for extremely preterm infants; however, MRI-based reference values for ventricular size at term-equivalent age are sparse.

**Objective:**

To present supratentorial ventricular size around term-equivalent age using MRI-based linear- and approximate volumetric measurements in extremely premature infants with and without germinal matrix-intraventricular haemorrhages, to assess whether ventricular size increases with haemorrhage presence and severity, and to determine which linear measurement best predicts volume of the lateral ventricles.

**Materials and methods:**

In total, 119 infants born before 28 gestational weeks (mean chronological age at MRI 14.6 weeks) were prospectively included and categorised as having either no haemorrhage or germinal matrix-intraventricular haemorrhages based on cerebral ultrasound findings in the neonatal period. Brain MRI was performed around term-equivalent age. Linear measurements and approximate volumetric measurements of ventricular size were obtained.

**Results:**

Infants with germinal matrix-intraventricular haemorrhages grade 4 had significantly larger supratentorial ventricular systems compared to those with no haemorrhage or grade 1, including both linear measurements and approximate volumetric measurements. No differences were observed between infants with no haemorrhage and grades 1 or 2. Bilateral haemorrhages resulted in larger ventricular sizes than unilateral haemorrhages. Frontal horn depth and thalamo-occipital distance demonstrated the strongest correlations with lateral ventricle volume.

**Conclusion:**

Supratentorial ventricular size around term-equivalent age varies with severity and laterality of neonatal germinal matrix-intraventricular haemorrhages, with grade 4 associated with the largest ventricles. Frontal horn depth and thalamo-occipital distance were the best linear predictors of lateral ventricular volume.

**Graphical Abstract:**

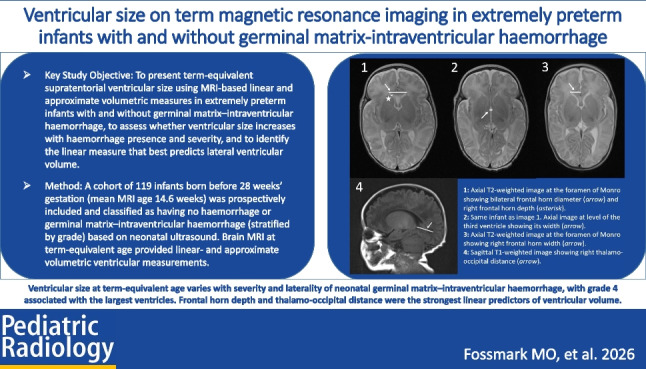

## Background

Infants born preterm, defined as prior to 37 weeks of gestation, tend to have larger cerebral ventricles at term-equivalent age compared to term-born infants [1, 2]. Among preterm infants, those born extremely preterm (before 28 weeks of gestation) exhibit even larger cerebral ventricles than those born at later preterm gestational ages [3]. Important pathologies contributing to this phenomenon are germinal matrix-intraventricular haemorrhages [4, 5] and white matter injury [6–9]. Germinal matrix-intraventricular haemorrhages are the most common intracranial haemorrhage in premature infants, with the highest risk observed in those with the lowest gestational age and birth weight [5, 10, 11]. The reported global incidence of these haemorrhages in extremely premature infants varies greatly, ranging from 7–72% [12]. In their hallmark paper published in1978, Papile et al. [13] presented a grading system for germinal matrix-intraventricular haemorrhages based on severity, where grade 1 is confined to the subependymal germinal matrix (classically seen in the caudothalamic groove), grade 2 consists of intraventricular haemorrhage within normally-sized ventricles, grade 3 is defined by haemorrhage filling more than 50% of the ventricle with ventricular dilatation, and grade 4 constitutes a parenchymal periventricular haemorrhagic infarction [13]. Grades 1 and 2 are commonly referred to as mild germinal matrix-intraventricular haemorrhages, whereas grades 3 and 4 are considered severe [4, 5, 12, 14, 15]. Severe germinal matrix-intraventricular haemorrhage is associated with a higher risk of developing post-haemorrhagic ventricular dilatation [16].

Technological advancements in recent decades have enabled faster magnetic resonance imaging (MRI) acquisition, making brain MRI at term-equivalent age increasingly feasible for evaluating cerebral development and injury in preterm infants [17]. Compared to cranial ultrasound, MRI offers superior sensitivity for detecting subtle abnormalities [18]. According to a recent study, an MRI at term-equivalent age can provide useful information on long-term neuropsychological outcomes at school-age in children born extremely premature, having no severe disability [19]. Others have advocated its use in high-risk infants with intracranial pathology previously identified through cranial ultrasound [17, 20, 21]. Given this, it is reason to believe that MRI at term-equivalent age will become more widely used, warranting robust, MRI-based data on ventricular size.

The present study aims at contributing to the current body of knowledge by presenting supratentorial ventricular size around term-equivalent age in a cohort of extremely preterm infants, both with and without germinal matrix-intraventricular haemorrhages, using MRI-based linear and approximate volumetric measurements. We assess whether ventricular size increases with the presence and severity of germinal matrix-intraventricular haemorrhages and identify which linear measurement best predicts lateral ventricular volume. Additionally, the association between the presence of germinal matrix-intraventricular haemorrhages identified on ultrasound in the neonatal period and haemorrhagic sequelae detected on MRI around term-equivalent age is presented.

## Materials and methods

This study is part of Baby-Project Extreme Prematurity (PEP) [22], a prospective, population-based study examining outcomes in extremely preterm infants. From October 1, 2010, to December 31, 2018, all women residing in Western Norway (Helse Vest) facing threatening delivery before 28 weeks of gestation and treated at Haukeland University Hospital were invited to participate. After parental consent, 178 infants were enrolled; four withdrew, and one was excluded due to incomplete clinical data, leaving 173 with clinical information. These infants underwent serial cranial ultrasounds from birth to discharge (day 2, day 7, and day 21, at GA 36 weeks and at term age), with efforts made to obtain brain MRIs at term-equivalent age. For this analysis, only infants with both ultrasound and MRI data were included; thus, two infants were excluded due to missing ultrasound data. The final cohort comprised 119 extremely preterm infants. Prevalence data on subdural haemorrhages from this same cohort has previously been reported [23].

Of the 119 infants, 110 underwent MRI at term-equivalent age (40±2 weeks post-menstrual), with a mean chronological age of 14.3 weeks (range, 11.9–18.0). The remaining nine had MRIs between 37.8-weeks and 49.3-weeks post-menstrual age. Across all infants, the mean age at MRI was 14.6 weeks (range, 10.3–23.6). Delivery modes included caesarean section (60 infants, 50.4%), vaginal without pre-labour contractions (36, 30.2%), and vaginal with contractions (23, 19.3%). All infants received multiple cranial ultrasounds during hospitalisation.

### Cranial ultrasound assessment and classification

Cranial ultrasounds were performed according to a standardised protocol, by one of four consultant paediatric radiologists with more than 10 years of experience in head ultrasound imaging, using a Philips iU22 Ultrasound System with a 3–10-MHz convex transducer (Philips Healthcare, Amsterdam, Netherlands). In a later session, based on the most severe neonatal ultrasound findings, the infants were grouped into (a) no intracranial haemorrhage or (b) presence of germinal matrix-intraventricular haemorrhage. Group b was further classified using the Papile grading system [13]. Signs of cystic periventricular leukomalacia were also recorded.

### Magnetic resonance imaging examination and assessment

MRI scans were conducted on a 1.5-T Siemens MAGNETOM scanner with a head coil (Siemens Healthineers, Erlangen, Germany), using a “feed-and-wrap” technique and a predefined protocol (Table 1). Susceptibility-weighted imaging (SWI) was performed in 98 of 119 cases. Haemorrhagic sequelae were identified by signal loss on SWI and corresponding hypointensities on T2-weighted images. Structural changes, such as cysts or tissue loss, were also assessed when present.

**Table 1:** Sequences and technical parameters of the magnetic resonance imaging protocol

Sequence	Acquired voxel size (mm^3^)	Slice thickness (mm)	Slice gap (mm)	Repetition time (ms)	Echo time (ms)	Scan time (min)
Axial T2 turbo spin echo^a^	0.8×0.8×4.0	4.0	0.4	4,000.0	118.0	2:14
Sagittal T1 spin echo^b^	0.4×0.4×4.0	4.0	0.4	450.0	8.4	2:57
Axial T1 true inversion recovery	0.8×0.8×4.0	4.0	0.4	4,500.0	57.0	2:38
Axial diffusion-weighted imaging	1.2×1.2×4.0	4.0	0.4	4,800.0	96.0	2:30
Axial susceptibility-weighted imaging	0.8×0.8×2.0	2.0	0.4	49.0	40.0	3:49

^a,b^The sequences were adjusted using sequences with motion correction or rapid sequences when necessary

**Fig. 1:**
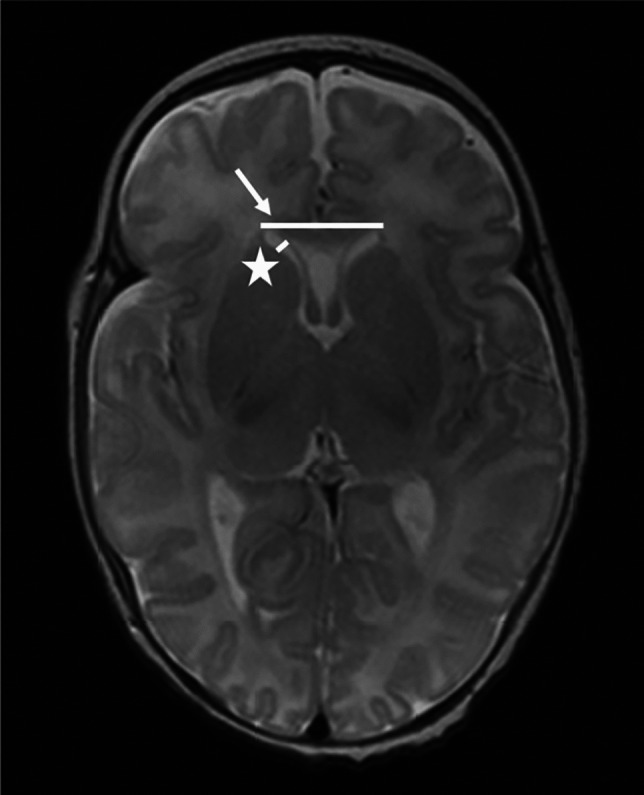
A 16-week-old girl born extremely premature at 25 gestational weeks with magnetic resonance imaging performed at term-equivalent age. Axial T2-weighted image without contrast at the level of the foramen of Monro shows the diameter of both frontal horns (*arrow*) and right frontal horn depth (*asterisk*). The infant had no intracranial haemorrhage or other pathology identified on cranial ultrasound in the neonatal period

**Fig. 2:**
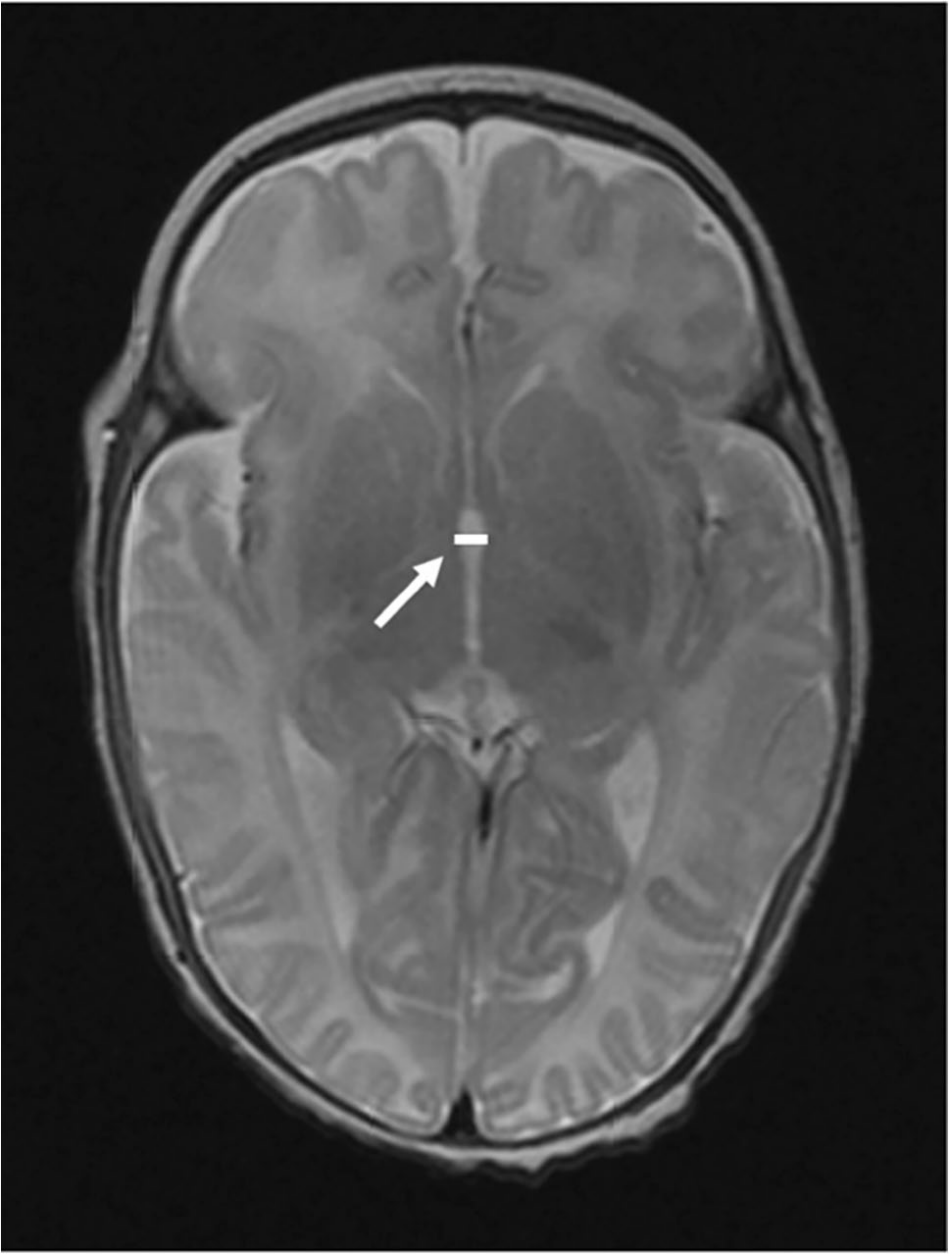
A 16-week-old girl born extremely premature at 25 gestational weeks with magnetic resonance imaging performed at term-equivalent age. Axial T2-weighted image without contrast at the level of the third ventricle shows third ventricle width (*arrow*). The infant had no intracranial haemorrhage or other pathology identified on cranial ultrasound in the neonatal period

**Fig. 3:**
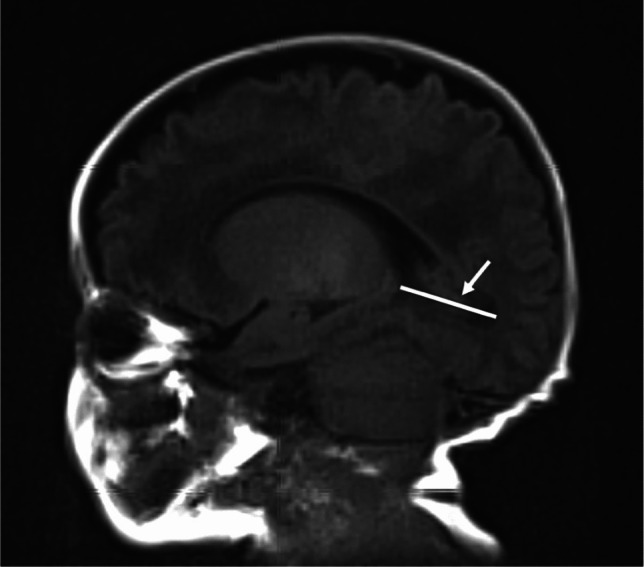
A 15-week-old boy born extremely premature at 26 gestational weeks with magnetic resonance imaging performed at term-equivalent age. Sagittal T1-weighted image without contrast shows thalamo-occipital distance on the right side (*arrow*). The infant had no intracranial haemorrhage or other pathology identified on cranial ultrasound in the neonatal period

**Fig. 4:**
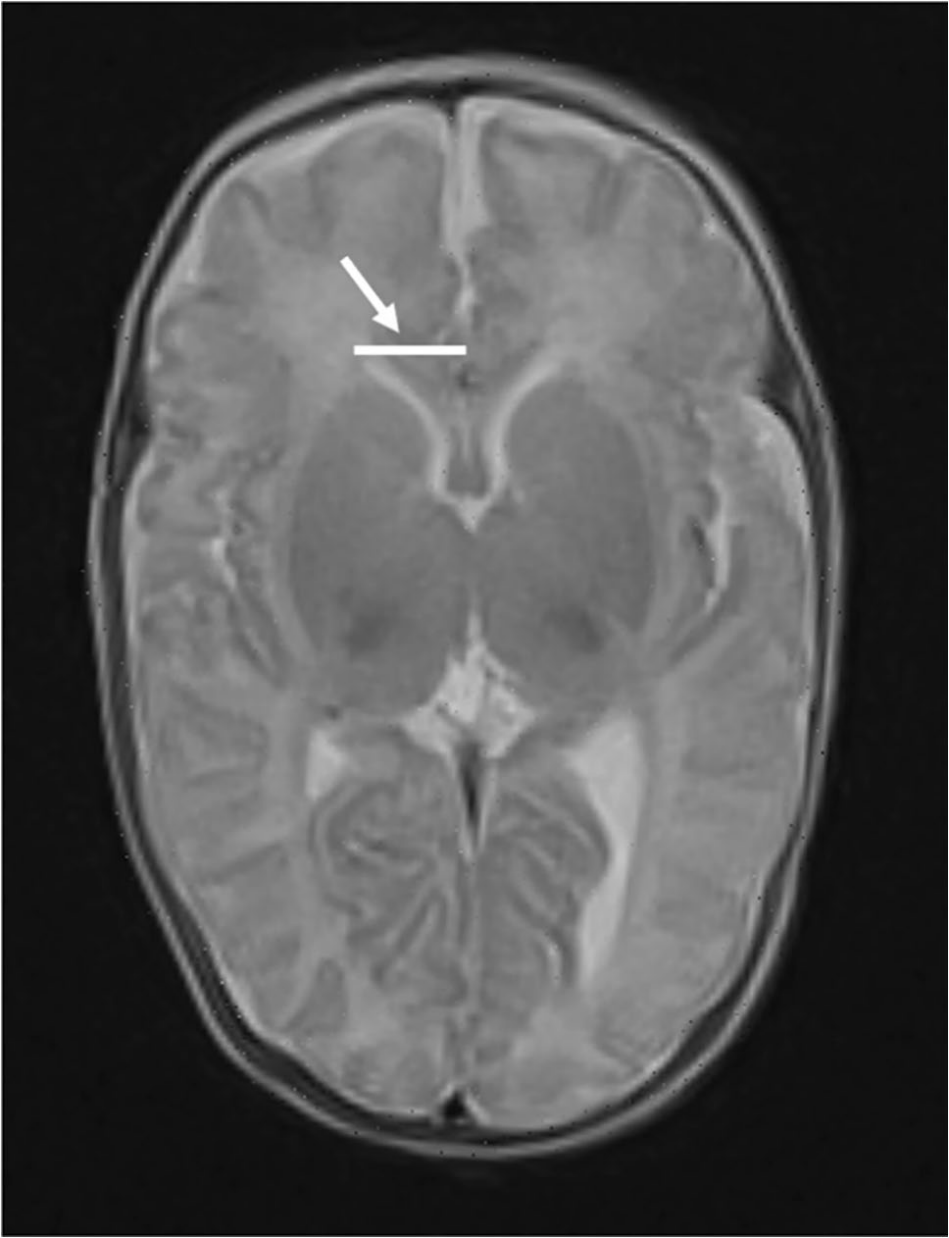
A 12-week-old girl born extremely premature at 28 gestational weeks with magnetic resonance imaging performed at term-equivalent age. Axial T2-weighted image without contrast at the level of the foramen of Monro shows frontal horn width on the right side measured in the axial plane (*arrow*). The infant had no intracranial haemorrhage or other pathology identified on cranial ultrasound in the neonatal period

Linear measurements of the supratentorial ventricles were taken from axial T2- and sagittal T1-weighted images. These included frontal horn depth and diameter at the level of the foramen of Monro (Fig. 1), third ventricle width (Fig. 2), thalamo-occipital distance (Fig. 3), and frontal horn width at the level of the foramen of Monro (Fig. 4).

**Fig. 5:**
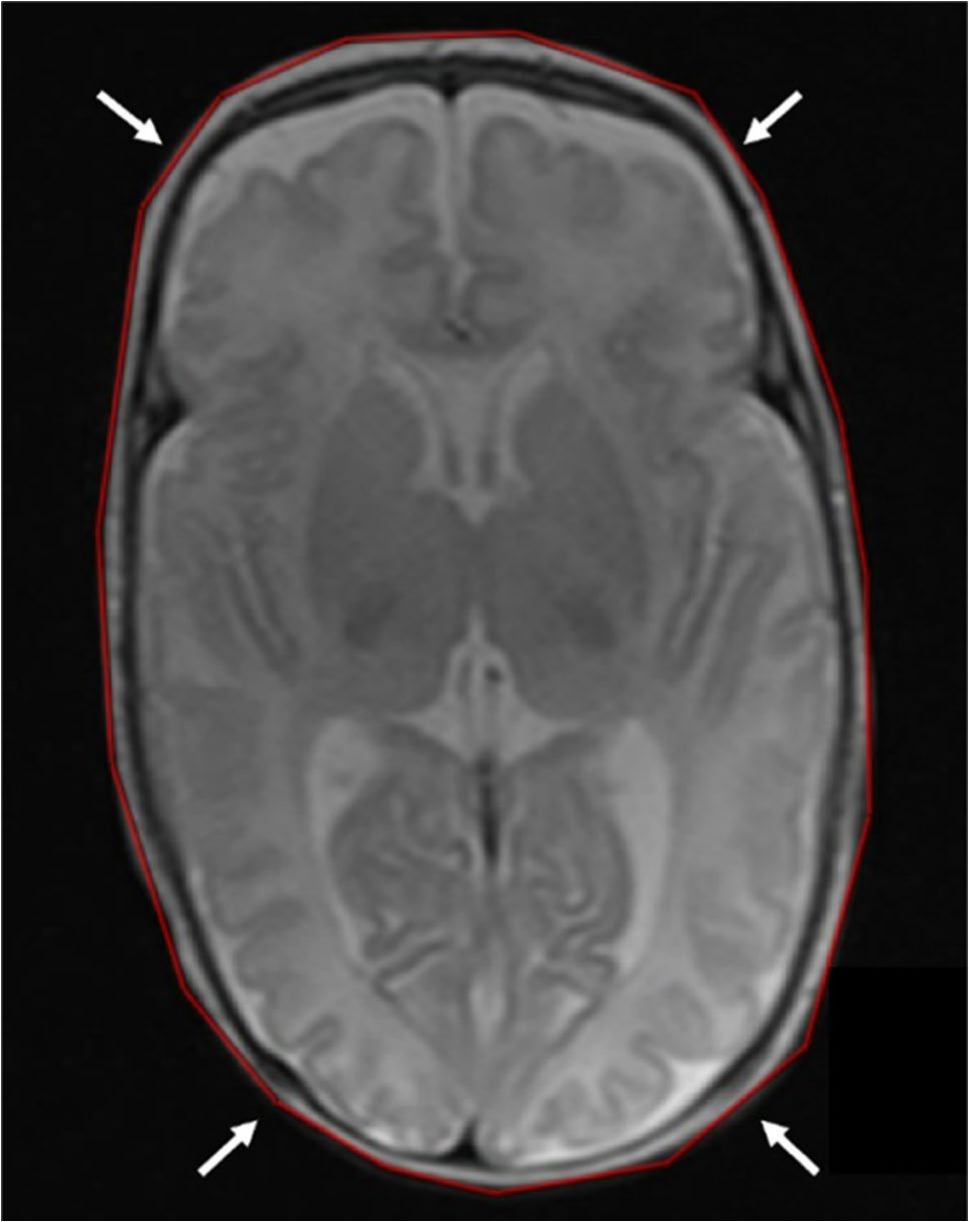
A 14-week-old girl born extremely premature at 27 gestational weeks with magnetic resonance imaging performed at term-equivalent age. Axial T2-weighted image without contrast demonstrates the measured head circumference, using multiplanar reconstructions, with measurement taken from the supraorbital bulge, encompassing the widest supra-auricular and occipital dimensions of the head (*arrows*). The infant had a left-sided germinal matrix-intraventricular haemorrhage grade 1 identified on cranial ultrasound performed at 7 days of age

As demonstrated by Rau et al. [24], head circumference can be reliably determined using measurements on three-dimensional (3-D) sequences with multiplanar reformations. Head circumference was measured on axial T2-weighted MRI using 3-D multiplanar reconstructions. The largest circumference was measured from the supraorbital bulge around the widest supra-auricular and occipital points (Fig. 5). Ratios between linear ventricular measurements and head circumference were calculated to adjust for individual head circumference.

Approximate ventricular volumes were calculated using ANALYZE 4.0 software (AnalyzeDirect, Overland Park, KS) as described by Argyropoulou et al. [25]. The “Auto Trace” function identified ventricular areas based on intensity thresholds, with manual adjustments as needed. Volumes were estimated by multiplying the measured areas by slice thickness plus gap across relevant slices.

### Statistical analysis

Continuous data were presented as means±standard deviation (SD) with 95% reference intervals (calculated as mean±1.96×SD). Independent *t*-tests were conducted to assess differences between the means of two independent groups for continuous, normally distributed data. Associations between ultrasound findings in the neonatal period and MRI findings around term-equivalent were evaluated using a chi-squared test. One-way ANOVA with post hoc tests was employed to identify which specific groups differed significantly for multiple independent categorical variables and a single continuous dependent variable. Group differences of linear measurement-head circumference ratios were analysed using one-way ANOVA. To determine which linear measurement best predicts ventricular volume, standard multiple linear regression was used. All statistical analyses were performed using IBM SPSS version 28 (IBM, Chicago, IL).

### Ethics

Approval for the study was granted by the Regional Ethics Committee of Western Norway (REK number 15573). Informed consent was given by the caregiver(s). Data collection and storage were conducted in compliance with the General Data Protection Regulation. The study was performed in accordance with the ethical standards as laid down in the 1964 Declaration of Helsinki.

## Results

In total, 119 infants (63 female) were included in the study. Demographic characteristics (Table 2) did not differ significantly between sexes, except for birth weight, which was significantly lower in males (*P*=0.04).

**Table 2:** Demographic data on 119 infants born extremely premature during the period 2010–2018

Parameters	Female (*n*=63)	Male (*n*=56)	*P*-value	Total (*n*=119)
Mean gestational age in weeks (SD), range	26.1 (1.2), 23.1–27.9.1.9	26.3 (1.3), 23.0–27.9.0.9	0.41	26.2 (1.2), 23.0–27.9.0.9
Mean birth weight in grams (SD), range	877.1 (202.6), 500.0-1.0.0.0.0.0,470.0	799.8 (204.8), 444.0-1.0.0.0.0.0,205.0	0.04	840.7 (206.4), 444.0-1.0.0.0.0.0,470.0
Mean chronological age at time of MRI examination in weeks (SD), range	14.8 (2.2), 10.3–23.6.3.6	14.4 (1.7), 12.0–21.8.0.8	0.26	14.6 (2.0), 10.3–23.6.3.6
Mean post-menstrual age at time of MRI examination in weeks (SD), range	40.5 (2.1), 31.8–49.3.8.3	40.4 (0.9), 39.1–44.6.1.6	0.77	40.5 (1.7), 31.8–49.3.8.3

*MRI* magnetic resonance imaging, *SD* standard deviation

Range corresponds to the minimum and maximum values observed

### Intracranial pathology as identified on cerebral ultrasound in the neonatal period

Sixty (50.4%) of the 119 included infants were diagnosed with germinal matrix-intraventricular haemorrhage on cranial ultrasound performed in the neonatal period, while 59 (49.6%) showed no evidence of haemorrhage. No infants had intracranial haemorrhages on cranial ultrasound that fell outside the category of germinal matrix-intraventricular haemorrhage. Regarding the presence of germinal matrix-intraventricular haemorrhages, no significant differences were observed between sexes (Pearson chi-square with *P*=0.23); thus, the results were pooled. Of the 60 infants with germinal matrix-intraventricular haemorrhages, 40 had unilateral involvement, with 20 cases affecting the right side (Table 3). In three of the 119 infants, periventricular cysts were reported, suggestive of cystic periventricular leukomalacia. One of these infants had cysts as the only pathological finding on ultrasound, whereas the other two had additional germinal matrix-intraventricular haemorrhages (one with grade 1 and one with grade 2, respectively). Periventricular hyperechogenicity was noted in six of the 119 infants, though no further details or aetiological suggestions were provided. All six of these infants also had germinal matrix-intraventricular haemorrhage, with four categorised as grade 3, one as grade 1, and one as grade 2. None of the included infants had cerebellar haemorrhages as identified on cranial ultrasound.

**Table 3:** Grades of germinal matrix–intraventricular haemorrhages as identified on cranial ultrasound performed in the neonatal period in 119 extremely premature infants, classified according to Papile

Grades of germinal matrix-intraventricular haemorrhage identified on cranial ultrasound, classified according to Papile	Number (% of total, *n*=119)
Any germinal matrix-intraventricular haemorrhage (grades 1–4)	60 (50.4%)
Unilateral haemorrhage (right or left side)	40 (33.6%)
Grade 1	31 (26.1%)
Grade 2	10 (8.4%)
Grade 3	8 (6.7%)
Grade 4	11 (9.2%)

### Association between germinal matrix-intraventricular haemorrhage identified on cerebral ultrasounds in the neonatal period and haemorrhagic sequelae detected on magnetic resonance imaging around term-equivalent age

A significant association was observed between germinal matrix-intraventricular haemorrhage identified on cranial ultrasound in the neonatal period and haemorrhagic sequelae detected on MRI around term-equivalent age (Pearson chi-square with *P*<0.001). Among the 59 infants without germinal matrix-intraventricular haemorrhage on cranial ultrasound, 10.1% demonstrated haemorrhagic sequelae on MRI. In comparison, haemorrhagic sequelae on MRI were present in 80.6% with a grade 1 haemorrhage, 90.0% with a grade 2 haemorrhage, and in all infants who had experienced grade 3 or grade 4 haemorrhages (Appendix 1).

### Magnetic resonance imaging-based linear measurements of supratentorial ventricular system

MRI-based linear measurements of the supratentorial ventricular system, stratified by the presence and severity of germinal matrix-intraventricular haemorrhage, are presented in Table 4. Significant group differences were observed for all linear measurements; thus, post hoc analyses were conducted (Appendix 2). Head circumference at term-equivalent age did not differ significantly between ultrasound groups (*P*=0.07).

**Table 4:** Magnetic resonance imaging-based linear ventricular measurements around term-equivalent age stratified by ultrasound findings in the neonatal period, 119 infants born extremely premature

MRI around term-equivalent age. Linear measurements. Mean (SD, 95% reference interval) in mm	Cerebral ultrasound from birth until discharge from hospital					
	No haemorrhage (*n*=59)	Germinal matrix-intraventricular haemorrhage classified according to Papile, based on the most severe grade (*n*=60)				
		Grade 1 (*n*=31)	Grade 2 (*n*=10)	Grade 3 (*n*=8)	Grade 4 (*n*=11)	*P*-value, *F*-test one-way ANOVA^a^
MR-based head circumference, cm	35.3 (1.4, 32.6–38.0.6.0)	35.0 (1.8, 31.5–38.5.5.5)	34.4 (1.8, 30.9–37.9.9.9)	35.5 (1.8, 32.0–39.0.0.0)	36.3 (1.4, 33.6–39.0.6.0)	0.07
Right frontal horn depth	2.3 (1.0, 0.3–4.3.3.3)	2.6 (0.9, 2.0–3.2.0.2)	2.4 (0.8, 1.9–2.9.9.9)	4.2 (2.0, 3.0–5.4.0.4)	3.8 (2.1, 2.5–5.1.5.1)	<0.01
Left frontal horn depth	2.5 (0.9, 0.7–4.3.7.3)	3.1 (1.1, 2.4–3.8.4.8)	2.7 (0.6, 2.3–3.1.3.1)	5.2 (2.1, 3.9–6.5.9.5)	5.0 (2.2, 3.6–6.4.6.4)	<0.01
Third ventricle width	2.6 (0.7, 1.2–4.0.2.0)	2.7 (0.6, 2.3–3.1.3.1)	2.7 (0.4, 2.5–2.9.5.9)	2.8 (1.3, 2.0–3.6.0.6)	4.0 (2.1, 2.7–5.3.7.3)	<0.01
Diameter both frontal horns	21.1 (3.1, 15.0–27.2.0.2)	20.7 (3.8, 18.3–23.1.3.1)	20.6 (3.1, 18.7–22.5.7.5)	25.1 (3.7, 22.8–27.4.8.4)	24.5 (3.4, 22.4–26.6.4.6)	<0.01
Right thalamo-occipital distance	25.4 (4.0, 17.6–33.2.6.2)	26.1 (4.4, 23.4–28.8.4.8)	24.7 (5.0, 21.6–27.8.6.8)	29.6 (5.4, 26.3–32.9.3.9)	30.7 (6.3, 26.8–34.6.8.6)	<0.01
Left thalamo-occipital distance	26.6 (4.3, 18.2–35.0.2.0)	28.1 (4.1, 25.6–30.6.6.6)	26.7 (3.2, 24.7–28.7.7.7)	31.7 (5.5, 28.3–35.1.3.1)	34.5 (7.6, 29.8–39.2.8.2)	<0.01
Right frontal horn width	11.3 (1.5, 8.4–14.2.4.2)	11.6 (1.6, 10.6–12.6.6.6)	12.1 (1.2, 11.4–12.8.4.8)	13.0 (2.1, 11.7–14.3.7.3)	13.3 (2.1, 12.0–14.6.0.6)	<0.01
Left frontal horn width	10.8 (1.5, 7.9–13.7.9.7)	11.4 (1.9, 10.2–12.6.2.6)	11.5 (1.0, 10.9–12.1.9.1)	12.5 (2.5, 11.0–14.0.0.0)	13.1 (1.8, 12.0–14.2.0.2)	<0.01

*MRI* magnetic resonance imaging, *SD* standard deviation

^a^Specific group differences are detailed in Appendix 2

Infants with grade 4 haemorrhage had significantly larger ventricular measurements compared to those with normal ultrasounds or grade 1 haemorrhage. No significant differences were found between the non-haemorrhage group and those with germinal matrix-intraventricular haemorrhage grade 1 or 2, nor between grades 1 and 2. However, among infants with germinal matrix-intraventricular haemorrhage, frontal horn depth was bilaterally greater in those with grade 3 compared to grades 1 and 2.

Among the 60 infants with germinal matrix-intraventricular haemorrhage, those with bilateral haemorrhages had significantly larger ventricular measurements for all parameters except third ventricle width (Table 5). In a subgroup analysis of infants with grade 1 haemorrhage (*n*=31), bilateral haemorrhages were associated with significantly larger right and left frontal horn depths, left frontal horn width, and third ventricle width. No significant differences were found for frontal horn diameters, right frontal horn width, or thalamo-occipital distances.

**Table 5:** Magnetic resonance imaging-based linear measurements of supratentorial ventricular system in 60 infants with germinal matrix-intraventricular haemorrhage, grouped by uni- or bilateral haemorrhages

MRI around term-equivalent age. Linear measurements. Mean (SD, 95% reference interval) in mm	Infants with unilateral haemorrhage (*n*=40)	Infants with bilateral haemorrhage (*n*=20)	*P*-value, independent *t*-test
Right frontal horn depth	2.5 (1.2, 0.2–4.9.2.9)	3.8 (1.6, 0.8–6.9.8.9)	<0.01
Left frontal horn depth	3.0 (1.3, 0.4–5.6.4.6)	4.9 (1.8, 1.3–8.5.3.5)	<0.01
Third ventricle width	2.7 (0.7, 1.3–4.1.3.1)	3.5 (1.7, 0.1–6.9.1.9)	0.06
Diameter both frontal horns	20.4 (3.6, 13.4–27.4.4.4)	24.6 (3.8, 17.2–32.1.2.1)	<0.01
Right thalamo-occipital distance	26.1 (4.5, 17.2–35.0.2.0)	29.3 (6.3, 17.0–41.6.0.6)	0.03
Left thalamo-occipital distance	28.4 (4.1, 20.3–36.5.3.5)	31.7 (7.4, 17.2–46.2.2.2)	0.03
Right frontal horn width	11.7 (1.6, 8.7–14.8.7.8)	13.0 (1.9, 9.3–16.8.3.8)	<0.01
Left frontal horn width	11.3 (1.6, 8.1–14.5.1.5)	13.0 (2.0, 9.2–16.9.2.9)	<0.01

*MRI* magnetic resonance imaging, *SD* standard deviation

### Adjustment of linear measurements to head circumference

One-way ANOVA revealed significant *F*-test results across all group comparisons for linear measurement-to-head circumference ratios, warranting post hoc analysis. Most group differences in linear ventricular measurements remained statistically significant after adjusting for head circumference, notably for the no-haemorrhage vs. germinal matrix-intraventricular haemorrhage grade 3 and 4 groups, where most ratios remained significant. Nevertheless, some initially significant differences became non-significant post-adjustment. These included mostly ratios in the germinal matrix-intraventricular haemorrhage grade 1 vs. grade 4 group, in addition to some ratios in the grade 1 vs. grade 3 group. For third ventricle width, significant differences between the no haemorrhage group and germinal matrix-intraventricular haemorrhage grade 3, as well as between grades 1 and 3, emerged only after adjustment (Appendix 3).

### Magnetic resonance imaging-based approximate volumetric measurements of supratentorial ventricular system

In total, 115 MRI examinations were judged of sufficient quality for assessment of volumes of lateral ventricles and total supratentorial ventricular system, while 109 examinations were included for assessment of the third ventricle volume. Approximate ventricular volumes by neonatal ultrasound group are shown in Table 6. One-way ANOVA revealed significant group differences, warranting post hoc analysis (Appendix 4).

**Table 6:** Magnetic resonance imaging-based approximate volumetric measurements around term-equivalent age by ultrasound findings in the neonatal period; 115 infants born extremely premature

MRI around term-equivalent age. Volumetric measurements. Mean (SD) in mm^3^	Cerebral ultrasound from birth until discharge from hospital					
	No haemorrhage	Germinal matrix-intraventricular haemorrhage classified according to Papile, based on the most severe grade				
		Grade 1	Grade 2	Grade 3	Grade 4	*P*-value, *F*-test one-way ANOVA^a^
Right lateral ventricle	5,412.4 (5,203.0)	5,554.7 (3,258.3)	5,185.3 (1,633.4)	11,584.5 (13,981.9)	18,341.3 (24,669.9)	<0.01
Left lateral ventricle	6,055.1 (4,473.5)	6,907.5 (3,634.3)	6,099.3 (1,813.1)	13,098.5 (12,030.6)	22,751.8 (32,014.4)	<0.01
Third ventricle	321.9 (106.0)	309.4 (125.2)	276.0 (135.7)	369.9 (153.4)	716.1 (983.7)	<0.01
Total supratentorial ventricular system	11,766.0 (7,550.3)	12,751.1 (6,287.3)	11,560.6 (3,352.0)	25,052.9 (25,873.3)	41,809.2 (56,010.4)	<0.01

*MRI* magnetic resonance imaging*, SD* standard deviation

^a^Specific group differences are detailed in Appendix 2

Infants with germinal matrix-intraventricular haemorrhage grade 4 had significantly larger ventricular volumes than those with no haemorrhage or with grades 1 or 2. No other group comparisons reached significant findings, including those involving grade 3 (Appendix 4).

For the right lateral ventricle, right frontal horn depth (*β*=0.41, *P*<0.01) and right thalamo-occipital distance (*β*=0.33, *P*<0.01) were significant predictors of volume, while frontal horn width was not (*β*=0.11, *P*=0.23). On the left, thalamo-occipital distance (*β*=0.46, *P*<0.01) and frontal horn depth (*β*=0.41, *P*<0.01) were also significant, but frontal horn width was not (*β*=–0.07, *P*=0.39). Variance inflation factors (VIF<2.0) indicated no multicollinearity. Overall, frontal horn depth and thalamo-occipital distance were stronger predictors of lateral ventricular volume than frontal horn width, suggesting they may be more reliable markers of ventricular size on MRI in this population.

## Discussion

This study shows that supratentorial ventricular size around term-equivalent age in infants born extremely premature varies with the severity and laterality of neonatal germinal matrix-intraventricular haemorrhage, with grade 4 being associated with the largest ventricles. No differences in ventricular size were observed between infants with no haemorrhage and mild germinal matrix-intraventricular haemorrhage (grades 1 and 2). Bilateral haemorrhages resulted in larger ventricles than unilateral haemorrhages. Frontal horn depth and thalamo-occipital distance demonstrated the strongest correlations with lateral ventricle volume. To our knowledge, there are currently no other population-based studies that report MRI-based supratentorial ventricular size in extremely premature infants at term-equivalent age stratified by both presence and degree of germinal matrix-intraventricular haemorrhages.

MRI offers significant advantages over cranial ultrasound in detecting cerebral injury, particularly in identifying white matter injury, a common concern in prematures [26–28]. An increasing body of evidence supports routine use of cerebral MRI at term-equivalent age as an integral component of clinical care for this patient group, particularly not only in high-risk infants with intracranial pathology previously identified through cranial ultrasound [17, 20, 21], but also in children without severe disabilities in childhood [19]. Several institutions have successfully established imaging protocols that consistently yield high-quality images from non-sedated infants, typically using the “feed-and-wrap” technique, thereby eliminating the risks and costs associated with sedation [29–32]. As a result, MRI performed at term-equivalent age is expected to play an increasingly important role going forward, and MRI-based data on ventricular size in this population is warranted.

Linear MRI-based measurements of ventricular size at term-equivalent age in prematures have been reported by Buchmayer et al. [33] and Naud et al. [34]. Buchmayer et al. [33] excluded infants with major cranial MRI abnormalities (including germinal matrix-intraventricular haemorrhage ≥grade 2) and found mean frontal horn widths of 11.4–13.1 mm, frontal horn depths of 3.2–4.0 mm, and third ventricle widths of 3.1–4.0 mm, varying by gestational age and scan timing. They also noted an inverse correlation between gestational age and ventricular size. Naud et al. [34] included infants born before 32 weeks of gestation, excluding only those with genetic syndromes or brain malformations. They reported a mean frontal horn depth of 2.54 mm. Findings from both studies align well with our results.

Several studies have reported MRI-based measurements of ventricular volume, particularly lateral ventricles, in preterm infants at term-equivalent age [35–39]. Storbeck et al. [35] studied 73 infants born before 32 weeks or under 1,500 g and found mean lateral ventricle volumes ranging from 2,894–3,406 mm^3^, with higher volumes associated with germinal matrix-intraventricular haemorrhage, especially on the left (*P*=0.02), though severity was not stratified. Bengtsson et al. [36] reported mean volumes of 5,400 mm^3^ (left) and 4,300 mm^3^ (right) in 34 preterm infants, noting strong correlations between MRI and ultrasound, particularly with regard to the frontal horns, but without haemorrhage-specific analysis. Toppe et al. [38] found average volumes of 3,508 mm^3^ (left) and 2,957 mm^3^ (right) in 67 preterm infants, identifying germinal matrix-intraventricular haemorrhage as an independent predictor of volume, without grading of the haemorrhages. Sheng et al. [39] analysed 194 infants and reported a median ventricular volume of 6,600 mm^3^ (IQR 4,800–9,100 mm^3^); although germinal matrix-intraventricular haemorrhage grades were recorded, volumes were not stratified by severity. Finally, Beijst et al. [37] studied 31 preterm infants with germinal matrix-intraventricular haemorrhage and reported a wide range in total ventricular volume (mean 8,100±5,900 mm^3^; range 2,000–30,300 mm^3^), also showing significant correlations between MRI-based volumes and ultrasound measurements such as frontal horn width, depth, and thalamo-occipital distance.

In our study, we found mean volumes of 5,412.4±5,203.0 mm^3^ and 6,055.1±4,473.5 mm^3^ for the right and left ventricles, respectively, in infants with normal ultrasound examinations during the neonatal period. The mean lateral ventricular volume was larger in infants with germinal matrix-intraventricular haemorrhage. These values exceed those reported by Storbeck et al. [35] and Toppe et al. [38], which in part can be attributed to their inclusion of infants born up to 32 and 34 gestational weeks, whilst we included infants born prior to 28 gestational weeks. Our numbers better compare to the numbers reported by Bengtsson et al. [36] and Sheng et al. [39]. Unlike previous studies, our analysis included measurements of third ventricle volume in addition to lateral ventricle volume. Furthermore, we provide a detailed assessment of ventricular size, stratified not only by the presence of intracranial pathology but also by varying degrees of germinal matrix-intraventricular haemorrhage. Severe germinal matrix–intraventricular haemorrhage (≥grade 3) is associated with an increased risk of post-haemorrhagic ventricular dilatation, particularly in grade 3 cases [16, 40, 41]. In our cohort, with MRI performed around term-equivalent age, both grade 3 and grade 4 haemorrhages were associated with larger ventricles compared with infants with no haemorrhage or grade 1–2 haemorrhage. This aligns with previous reports and is likely explained by the presence of post-haemorrhagic ventricular dilatation in some of these infants. Like Beijst et al. [37], we found that frontal horn depth and thalamo-occipital distance showed high correlation with lateral ventricle volume.

In infants with grade 4 haemorrhage (periventricular haemorrhagic infarction), delineation of the ventricular borders can be challenging, as the porencephalic cysts often communicate with the ventricular system and their walls become continuous with the ventricular lining. In this context, separating the cystic component from the ventricles is anatomically arbitrary, and inclusion of these spaces within the measured ventricular volume may better represent the extent of the underlying pathology. Thus, porencephalic cysts were included in the ventricular volume assessment in infants with grade 4 haemorrhage and may to some extent contribute to the increased volume in this group. In lower germinal matrix-intraventricular haemorrhage grades, ventricular enlargement likely reflects different mechanisms, such as post-haemorrhagic dilatation [4, 42, 43] or tissue volume loss without cystic transformation [44].

The strengths of this study include its prospective, population-based design including both infants with and without germinal matrix-intraventricular haemorrhage, the inclusion of a relatively high number of extremely premature infants, and the use of meticulous image analysis in combination with state-of-the-art MRI techniques. Nevertheless, several limitations must be acknowledged. Head circumference was measured using multiplanar reconstructions on T2-weighted MR images due to the absence of clinical measurements, which may have introduced measurement error. Regarding the approximate volumetric measurements, five MR examinations for lateral ventricular volume and 11 for third ventricular volume were excluded due to motion artefacts. The volumetric analysis was further limited by large standard deviations, likely due to high variability in ventricular volume in this population. The use of the ANALYZE 4.0 software, which relies on intensity thresholding and semi-automated region tracing, may introduce operator-dependent variability and is susceptible to partial volume effects and suboptimal delineation, especially in the presence of motion artefacts or poor contrast. Moreover, the approach used to estimate volumes (multiplying cross-sectional areas by the sum of slice thickness and interslice gap) assumes uniform geometry and may not accurately represent the complex morphology of the ventricular system. Finally, although we found a significant association between neonatal haemorrhage and MRI-detected sequelae at term-equivalent age, the use of the “feed-and-wrap” technique led to failed scans in some cases, with absence of successful SWI sequences in 23 infants. Further, using a slice thickness of 4 mm with gaps of 0.4 mm may have resulted in missed subtle haemorrhagic lesions.

## Conclusion

We present MRI-based supratentorial ventricular size at term-equivalent age in infants born extremely premature, stratified by the presence and severity of germinal matrix-intraventricular haemorrhage identified in cranial ultrasound in the neonatal period. Half of the infants had germinal matrix-intraventricular haemorrhage on cranial ultrasounds, of which the majority were grade 1. Despite no significant differences in head circumference, MRI-based ventricular size around term-equivalent age varies with the severity and laterality of the haemorrhages in the neonatal period, with grade 4 being associated with the largest ventricles. Among the linear measurements taken, frontal horn depth and thalamo-occipital distance were found to be the best predictors of lateral ventricle volume at term-equivalent age. There was a high association between the presence of germinal matrix-intraventricular haemorrhage on cranial ultrasound in the neonatal period and presence of haemorrhagic sequelae detected on brain MRI around term-equivalent age.

## Data Availability

No datasets were generated or analysed during the current study.

## Appendix

See Appendix Table 7, Table 8, Table 9 and Table 10.

**Table 7:** Cerebral sequelae as identified on MRI around term-equivalent age in 119 ex-premature infants stratified by findings on cerebral ultrasound during the neonatal period

Brain MRI around term-equivalent age	Cerebral ultrasound from birth until discharge from hospital						
	No haemorrhage (*n* = 59)	Germinal matrix-intraventricular haemorrhage classified according to Papile, based on the most severe grade (*n* = 60)					
		Grade 1 (*n* = 31)		Grade 2 (*n* = 10)	Grade 3 (*n* = 8)	Grade 4 (*n* = 11)	Total:
No haemorrhagic sequelae (*n* = 60)	53	6	1		0	0	60
Isolated germinal matrix haemorrhage without porencephalic cyst (*n* = 40)	5	19	8		4	3	39
Isolated germinal matrix haemorrhage, with porencephalic cyst (*n* = 4)	0	1	0		0	3	4
Isolated cerebellar haemorrhage (*n* = 2)	1	1	0		0	0	2
Germinal matrix haemorrhage, without porencephalic cyst, with cerebellar haemorrhage (*n* = 10)	0	4	1		4	1	10
Germinal matrix haemorrhage, with porencephalic cyst, with cerebellar haemorrhage (*n* = 4)	0	0	0		0	4	4
Total:	59	31	10		8	11	119

*MRI* magnetic resonance imaging

**Table 8:** One-way ANOVA with post hoc test, *P*-values, linear ventricular measurements

Comparison	Right frontal horn depth	Left frontal horn depth	Third ventricle width	Diameter frontal horns	Right thalamo-occipital distance	Left thalamo-occipital distance	Right frontal horn width	Left frontal horn width
No haemorrhage vs. grade 1	0.86	0.36	1.00	1.00	0.97	0.71	0.91	0.65
No haemorrhage vs. grade 2	1.00	1.00	1.00	1.00	0.99	1.00	0.59	0.82
No haemorrhage vs. grade 3	<0.01	<0.01	0.99	0.02	0.12	0.05	0.04	0.08
No haemorrhage vs. grade 4	<0.01	<0.01	<0.01	0.03	<0.01	<0.01	<0.01	<0.01
Grade 1 vs. grade 2	1.00	0.92	1.00	1.00	0.92	0.92	0.92	1.00
Grade 1 vs. grade 3	<0.01	<0.01	1.00	0.02	0.29	0.30	0.19	0.45
Grade 1 vs. grade 4	0.05	<0.01	<0.01	0.03	0.04	<0.01	0.03	0.04
Grade 2 vs. grade 3	<0.01	<0.01	1.00	0.06	0.16	0.17	0.75	0.72
Grade 2 vs. grade 4	0.09	<0.01	0.02	0.09	0.02	<0.01	0.44	0.20
Grade 3 vs. grade 4	0.92	1.00	0.04	1.00	0.98	0.71	1.00	0.94

Grades refer to the degree of germinal matrix-intraventricular haemorrhage

**Table 9:** One-way ANOVA with post hoc test, *P*-values, linear ventricular measurement–to–head circumference ratio

Comparison of linear ventricular measurement–to–head circumference ratio	Right frontal horn depth	Left frontal horn depth	Third ventricle width	Diameter frontal horns	Right thalamo-occipital distance	Left thalamo-occipital distance	Right frontal horn width	Left frontal horn width
No haemorrhage vs. grade 1	0.82	0.28	0.82	1.00	0.91	0.38	0.73	0.36
No haemorrhage vs. grade 2	0.99	0.99	0.99	1.00	1.00	1.00	0.29	0.47
No haemorrhage vs. grade 3	<0.01^1^	<0.01^1^	<0.01^3^	0.02^1^	0.10	0.03^1^	0.07	0.10
No haemorrhage vs. grade 4	0.01^1^	<0.01^1^	0.01^1^	0.17^2^	0.02^1^	<0.01^1^	0.06^2^	<0.01^1^
Grade 1 vs. grade 2	1.00	0.93	1.00	1.00	0.97	0.94	0.81	0.99
Grade 1 vs. grade 3	<0.01^1^	<0.01^1^	<0.01^3^	0.02^1^	0.34	0.41	0.37	0.68
Grade 1 vs. grade 4	0.12^2^	<0.01^1^	0.12^2^	0.16^2^	0.15^2^	0.02^1^	0.38^2^	0.21^2^
Grade 2 vs. grade 3	0.02^1^	<0.01^1^	0.02	0.12	0.24	0.25	0.96	0.93
Grade 2 vs. grade 4	0.19	<0.01^1^	0.19^2^	0.44	0.12^2^	0.01^1^	0.98	0.64
Grade 3 vs. grade 4	0.82	0.99	0.82^2^	0.93	1.00	0.88	1.00	0.99

Grades refer to the degree of germinal matrix-intraventricular haemorrhage

^1^Group difference persisted as statistically significant after controlling for head circumference

^2^The initially observed group differences were no longer statistically significant after adjusting for head circumference

^3^Group differences emerged as statistically significant only after adjusting for head circumference

**Table 10:** One-way ANOVA with post hoc test, *P*-values, approximate volumetric measurements

Comparison	Right lateral ventricle volume	Left lateral ventricle volume	Third ventricle volume	Total supratentorial ventricular system
No haemorrhage vs. grade 1	1.00	1.00	1.00	1.00
No haemorrhage vs. grade 2	1.00	1.00	1.00	1.00
No haemorrhage vs. grade 3	0.44	0.48	1.00	0.42
No haemorrhage vs. grade 4	<0.01	<0.01	<0.01	<0.01
Grade 1 vs. grade 2	1.00	1.00	1.00	1.00
Grade 1 vs. grade 3	0.51	0.64	0.99	0.54
Grade 1 vs. grade 4	<0.01	<0.01	<0.01	<0.01
Grade 2 vs. grade 3	0.61	0.68	0.98	0.60
Grade 2 vs. grade 4	0.01	<0.01	0.02	<0.01
Grade 3 vs. grade 4	0.54	0.34	0.19	0.37

Grades refer to the degree of germinal matrix-intraventricular haemorrhage

## Declarations

The study was approved by the Regional Ethics Committee of Western Norway (*REK number 15573*) and was performed in accordance with the ethical standards as laid down in the 1964 Declaration of Helsinki. Informed consent was obtained by the caretaker(s).
